# Jump performance and hop function, kinesiophobia and return to sports are important prognostic factors for a subsequent injury after an anterior cruciate ligament reconstruction: A 2‐year follow‐up cohort study

**DOI:** 10.1002/ksa.70104

**Published:** 2025-10-27

**Authors:** Daniel Niederer, Matthias Keller, Wolf Petersen, Karl‐Friedrich Schüttler, Turgay Efe, Tobias Engeroff, David A. Groneberg, Christine Heinrich, Michael Behringer, Natalie Mengis, Andree Ellermann, Daniel Guenther, Georg Brandl, Björn Drews, Julian Mehl, Raymond Best, Lucia Pinggera, Christian Schoepp, Matthias Krause, Thomas Stein

**Affiliations:** ^1^ Institute of Occupational, Social and Environmental Medicine Goethe University Frankfurt Frankfurt am Main Germany; ^2^ OSINSTITUT Ortho & Sport Munich Germany; ^3^ Klinik für Orthopädie und Unfallchirurgie Berlin Germany; ^4^ Orthopaedicum Lich Giessen Gießen Germany; ^5^ Department of Sports Medicine and Exercise Physiology Goethe University Frankfurt Frankfurt am Main Germany; ^6^ Department of Orthopaedic Surgery and Traumatology Kantonsspital Baselland Basel Switzerland; ^7^ University of Basel Basel Switzerland; ^8^ Arcus Sportklinik Pforzheim Germany; ^9^ Department of Orthopaedic Surgery, Trauma Surgery, and Sports Medicine, Cologne Merheim Medical Center Witten/Herdecke University Witten Germany; ^10^ Department of Orthopaedic Surgery II Herz‐Jesu Krankenhaus Vienna Austria; ^11^ St. Vinzenz Clinic Allgäu Pfronten Germany; ^12^ Department for Orthopaedic Sports Medicine Klinikum rechts der Isar Munich Germany; ^13^ Department of Orthopaedic and Trauma Surgery Sportklinik Stuttgart Stuttgart Germany; ^14^ Department of Arthroscopic Surgery, Sports Traumatology and Sports Medicine BG Klinikum Duisburg gGmbH Duisburg Germany; ^15^ Department of Trauma and Reconstructive Surgery University Hospital Essen Essen Germany; ^16^ Department of Trauma, Hand and Reconstructive Surgery and Orthopaedic Surgery University Medical Center Hamburg‐Eppendorf Hamburg Germany; ^17^ SPORTHOLOGICUM – Knee Center Frankfurt ‐ Center for Sport and Joint injuries Frankfurt am Main Germany

**Keywords:** ACL, determinates, functional capacity, prediction, return to sports, RTS

## Abstract

**Purpose:**

Finding prognostic factors for a subsequent injury after an anterior cruciate ligament (ACL) reconstruction.

**Methods:**

We re‐analysed the data of two intervention studies on adults with a hamstrings or quadriceps tendon ACL reconstruction. All participants were prospectively monitored for 24 months. At the end of the individual postsurgery rehabilitation, numerous self‐reported and objective functional outcomes were quantified, all potential secondary injuries (primary outcome was the occurrence of secondary ipsi‐ or contralateral ACL injuries) and all rehabilitation and training measures were prospectively monitored. The association of potential factors with a secondary injury risk was determined using logistic mixed models.

**Results:**

We included 148 participants (mean age 25.3 years [standard deviation 5.1 years], 63 females). Eight participants had a subsequent ACL injury, among them seven ispilateral and one contralateral side ACL rupture. The final model for the likelihood of a subsequent ACL injury led to a sensitivity (correctly classified participants who had a subsequent ACL injury) of 83.3% and to a specificity (correctly identified participants without who did not have a subsequent ACL injury) of 100% (*n* = 93). The main contributing factors to subsequent ACL or any subsequent other injuries were: higher kinesiophobia values (odds ratio [OR] = 2.0, 95% confidence interval [CI] = 1.1–3.4), higher knee loading levels during activity (Tegner activity scale, OR = 29, 95% CI: 1.1–791), lower performance levels at the Balance front hop (OR = 0.13, 95% CI: 0.03–0.52), and higher dynamic valgus (knee separation distance in the frontal plane) during the landing of a drop jump landing (OR = 0.80, 95% CI: 0.65–0.98).

**Conclusion:**

Most of the predictive factors for a second subsequent injury after an ACL reconstruction are modifiable by adequate training and rehabilitation measures. The modification of these factors might decrease the secondary risk of injury risk.

**Level of Evidence:**

Level II, a prospective cohort study.

**Abbreviations**
ACL: anterior cruciate ligament
ACL‐RSI: RTS after ACL injury
BMI: body mass index
DRKS: German Clinical Trials Register
RCT: randomised controlled trial
RTS: return to sport
TSK: Tampa scale of kinesiophobia

## INTRODUCTION

After an index anterior cruciate ligament (ACL) rupture with subsequent autograft reconstruction, the risk for a subsequent second rupture is approximately 20% [25] and thus manyfold higher when compared to the risk of the index injury itself. Ipsilateral and contralateral ACL ruptures are equally common [25], and reducing the risk of a subsequent ACL injury is of great importance. Functional capacities play a crucial and role in assessing individuals' subsequent ACL injury risk [5, 10, 32]. Return to sport (RTS) decision‐making based on a shared decision process with particular focus on functional criteria can reduce subsequent ACL injury risk [3, 29]. It is important to note that not only test batteries as a whole, but also individual functional tests may have a predictive value for a subsequent ACL injury [2]. In particular strength, jump‐ and hop performance and self‐report function are named as the most important functional outcomes [2].

Functional capacity cannot be rated in isolation but must always be connected to further potential predictors of subsequent injuries; numerous potential factors are known in this context. From a sociodemographic and injury‐related perspective, sex/gender [4, 33], body mass index (BMI) [1, 4], age [4, 16], the time between injury and surgery [4, 33] and the type of autograft [33] are described in the literature as relevant risk factors for a subsequent ACL injury. Sport‐ and rehabilitation‐specific variables such as higher Tegner activity scales [33], the time to RTS and the level to which an athlete aims to return [1, 4, 33] as well as the extent of rehabilitative measures [16] may also contribute to an increased or decreased subsequent risk for reinjury after an index ACL rupture and reconstruction. Further self‐reported outcomes such as psychosocial readiness to RTS [1] and the amount of kinesiophobia [28] may also contribute to the risk of a subsequent injury. To effectively improve rehabilitation and prevent re‐injury, the contributions of potential factors to a second injury following an initial ACL rupture and reconstruction must first be identified and, where possible, modified.

Our prospective 2‐year follow‐up cohort study identified the contributions of these potential factors to the risk of a secondary ACL rupture, other subsequent knee injuries and any lower limb injuries following an initial ACL tear. We hypothesised that (1) lower exercise and rehabilitation volumes, (2) lower psychological readiness and lower functional outcomes as well as (3) traits and baseline factors such as athletic status increase the risk for secondary injuries.

## METHODS

### Design and ethical aspects

This cohort study represents a planned re‐analysis conducted as part of the PReP Project [21]. The data utilised in this analysis were originally collected from a randomised controlled trial (RCT) [20] and from an interventional matching cohort [22]. Independent institutional review board ethical approval was provided by the Ethics Committee of the Hessen Regional Medical Council on 27 June 2018 (reference approval no. FF 104/2017). The study was designed and conducted in agreement with the Declaration of Helsinki (Version Fortaleza 2013) and registered in the German Clinical Trials Register (DRKS): registration number DRKS00015313 (1 October 2018) (DRKS, drks.de; 01 October 2018).

### Participants

To be included in one of the underlying studies, individuals with an ACL rupture who had an appointment in one of the study centres were screened for eligibility by means of a structured informed consent process. All participants signed written and gave informed oral consent, and further provided consent to their data being re‐analysed in secondary analyses prior to enrolment. Inclusion criteria (to be included in one of the underlying studies) were: age between 18 and 36 years, presence of an acute unilateral ACL rupture and underwent (or being scheduled for) an arthroscopically assisted, anatomic ipsilateral quadriceps tendon, semitendinosus tendon, or semitendinosus‐gracilis tendon graft ACL reconstruction. Participants were included if they reported to be an active elite or recreational athlete of any type prior to the injury, and also aimed to return to their previous sporting activity.

Further exclusion criteria were: a meniscus lesion of larger than 2 cm, a cartilage lesion categorised higher than ICRS II°, any previous surgery on the contralateral leg, a leg malalignment (varus or valgus) greater than 5°, multiligament injury patterns and pregnancy.

### Study flow

**Figure 1:**
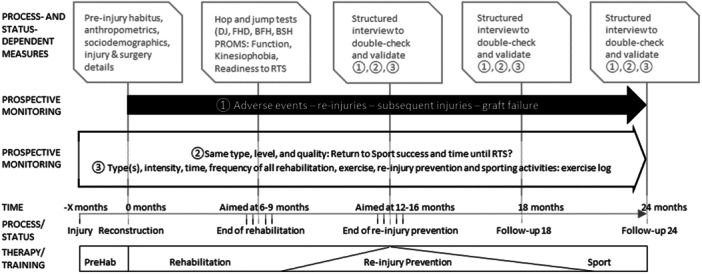
Study flow. All training and rehabilitation measures were process‐ and status‐dependent, accompanied by measurements consisting of all self‐report and functional outcomes. All other aspects, such as the potential RTS success, subsequent issues, re‐ or secondary injuries, and all rehabilitation and training measures, were prospectively monitored. BFH, balance front hop; BSH, balance side hop; DJ, drop jump; FHD, front hop for distance; PROMS, self‐reported outcomes; RTS, return to sport.

All participants were prospectively monitored until their individual 24‐month postreconstruction follow‐up. The detailed study flow is displayed in Figure 1. Preoperative rehabilitation was followed by reconstruction, which was followed by the postsurgery early‐, mid‐ and late‐stage rehabilitation and re‐injury prevention program. The corresponding points in time were individually set and accompanied by process‐ and status‐dependent measurements consisting of self‐report and functional outcomes. Prospective data were collected using structured, repetitive telephone interviews and detailed exercise logs. Data on preoperative rehabilitation, surgical reconstruction, postsurgery rehabilitation and re‐injury prevention intervention were prospectively collected. Potential return‐to‐sport success, as well as all rehabilitation and training measures, were monitored throughout the study's duration.

All functional and self‐reported outcomes were collected between the individually determined end of the postsurgery rehabilitation and the onset of the re‐injury prevention intervention (mean of 241 days, standard deviation 92 days postreconstruction). All self‐reported outcomes were completed online at www.soscisurvey.de using a standardised survey.

### Subsequent injuries

Both prospectively and during each contact (mostly during the assessments and telephone interviews), participants reported all adverse events. Particular focus was on secondary subsequent ACL injuries (ipsilateral and contralateral ACL tears). Nonetheless, all musculoskeletal conditions were monitored, irrespective of the location, whether they lead to a time loss in any performance or their severity. The date and mechanism of any secondary injury (contact, indirect contact, no contact, no acute trauma, chronic problems) were also monitored. The participants reported all subsequent ACL injuries, and any other all musculoskeletal condition shortly after their occurrence by telephone or online at www.soscisurvey.de. To doublecheck the incidents (and to reveal those which have not been reported yet), participants were asked for any previous incidence and/or secondary injury four times: at the end of the postsurgery rehabilitation measurement, at the end of the re‐injury‐prevention, and during two telephone interviews, one at the 18‐ and one at the 24‐month postreconstruction time point. The formal medical diagnosis of the incidence was noted.

### Potential contributors to the risk of a secondary injury

Baseline values and traits: Sociodemographic and anthropometric values (sex/gender, height, weight and age), injury mechanisms (contact‐free, indirect contact, contact), preinjury type(s) and level of sport and training volumes were asked early after surgery. The latter were used to classify the athletic status as elite‐ or nonelite, where elite status was defined as receiving assurance‐relevant payment for the sports performance. Further surgery‐specific outcomes such as the graft type and other relevant surgical information were retrieved from the pseudonymised surgery reports.

Training and rehabilitation measures protocol: All rehabilitation measures (type, duration, intensity and frequency) between injury and reconstruction, between reconstruction and the end of the formal rehabilitation and since the end of the formal rehabilitation have been monitored. To achieve this, all participants completed detailed exercise logs. The type (rehabilitation, sport type and exercise), frequency (times per week) and dose (minutes per week) of each session were noted [17]. The initial phase of the rehabilitation was the medically prescribed formal rehabilitation with a graduated transition to re‐injury preventive training. The initiation of the latter was aimed to commence at 9–12 months postreconstruction. It is important to note that some of the participants also exercised as part of the intervention trials within the PReP‐project: half of the hamstring graft participants were randomised and all quadriceps graft participants were allocated to a home‐based re‐injury prevention programme (Stop‐X) [21]. The comparator arm was usual care follow‐up treatment plus guideline recurrence prevention [21]. The training frequency for both arms was three times per week, with the aim of a duration of 30 min for each session. The usual care intervention consisted of nonsupervised impact exercises (Supporting Information S1: Table S1) and dynamic exercises in the frontal plane, followed by side‐cutting manoeuvres and concluding with dynamic multi‐directional stabilisation exercises. The Stop‐X intervention was partially supervised and completely stepwise graduated based on wound healing and, in particular, functional progression criteria. Basic (secondary) preventive strategies, running exercises/agility exercises, self‐perturbed postural control exercises, jumping exercises, plyometric and strengthening exercises were the main components of the Stop‐X‐programme. Please refer to the study protocol [21] and the Supporting Information S1: Table S1 for more details on this intervention.

Objective functional outcomes/Functional jump and hop tests: The functional measurements consisted of batteries of hop and jump tests. The drop jump screening test was followed by Balance front and side hops, as well as quantitative (cm) assessments of the Front hop for distance performance.

During the drop jump screening test, participants took a bipedal hip‐width stance on a box with a 32 cm target height while the hands had to be kept on the hips. A drop jump followed: frontal step—drop—reactive jump with the shortest possible ground contact time. The knee joint separation distance was rated at predefined points during this drop jump cycle: at the initial ground contact at the end of the drop from the box, and at the lowest point of the body's centre of gravity at the jump's reversal point (including the calculation of the transition = angle change in the frontal plane between these two landmarks). At each of these points, the distance between (1) the hip joints and (2) the middle of the two knees was measured, and a percentage of the knee distance in comparison to the hip distance was calculated (Kinovea, France) to build the normalised knee separation distance (which yielded the outcome). The normalisation approach allows for the elimination of a potential skewness of the smartphone position by the comparison of the knee separation distance to the hip width (which is in the same plane as the knees) [23].

For the landing quality rating, the Balance front hop and the Balance side hop test [12] were evaluated. Participants had to hop over a 40 × 40 cm square with their hands on their hips. The end position after landing had to be kept for at least 3 continuous seconds, during which the quality rating criteria were: (1) foot placement on the ground with whole sole support, foot remaining stable on the ground after landing (1 point); (2a) appropriate latero‐medial position control, knees remained in the sagittal plane, (2b) adequate and sufficient knee and hip flexion; (3a) no lateral trunk movements and (3b) aligned parallel to the lower leg, no excessive trunk flexion and the trunk remained in the sagittal and transversal planes [12]. The Balance hops display Kappa values of between 0.64 (balance front hop) and 0.79 (balance side hop), and the exact agreement between examiners is excellent (83%–93%) [11]. Two hops per trial and leg were performed, and the better attempt was selected for further analysis.

For the Front hop for distance (formally known as the Single leg hop for distance) [2, 9, 15], one hops as far as possible and must land in a controlled manner. The hopping distance was measured from toe at take‐off to heel at landing. The hands can be used for hop and landing control. Three hops per leg were performed in a randomised order. The best trial per leg (cm) was selected for further analysis. Measurement properties for the test are excellent (ICC = 0.97, confidence interval [CI]: 0.9–0.99); standard error of measurement 3.5% [14]). The cut‐off/threshold for the present case was selected to be an LSI of 90% for the Front hop for distance.

All jump and hop test were performed self‐administered while wearing (the same intraindividual) sport shoes and comparable clothing. Beyond that, all jump and hop tests were filmed from a frontal position (3 m distance) using the participants' own smartphone cameras. The videos were transferred using a safe form of large content transfer (PowerFolder Enterprise File Sync and Share; Germany) and were expert‐rated using the investigator‐blinded videos (drop jump screening test and Balance hops only). In cases of incorrect execution, the tests were repeated. This thorough smartphone camera‐based approach is valid when compared to 3D motion‐capture systems for the analyses of sagittal plane knee angles [6].

Self‐reported outcomes: To assess the psychological readiness to RTS, the participants completed the questionnaire ‘RTS after ACL injury’—(ACL‐RSI) [7]. This questionnaire assesses the psychological readiness of a person to RTS (by loading his/her reconstructed knee during a sporting activity). Ten 11‐point Likert scale questions are used, and a total score value of >56 points indicates sufficient confidence for RTS [8]. The sum score assessment is highly reliable (Cronbach's *α* = 0.92) [30] and valid [7].

The knee injury and osteoarthritis outcome score (KOOS) subscales of sport (SPORT), pain (PAIN), symptoms (SYMPTOMS) and activities of all daily living (ADL) subsequently assessed self‐reported knee function and symptoms [7]. The KOOS (items must be scored from 0 to 4) contains thresholds/cut‐offs for nonrestricted ratings (‘nonsymptomatic’): [19] PAIN 89 points, SYMPTOMS 83 points, ADL 95 points and SPORT 72 points.

Further self‐reported outcomes consisted of the Tegner activity scale (sporting activity level) and fear of movement (Tampa scale of kinesiophobia [TSK]) [7]. The Tegner activity scale [27] contains a 0–10 point Likert scale to assess a participant's activity level, scaled from low‐level daily living activity to high‐level competitive sports. Scoring is from 0 = low level activity regarding knee loading, up to and including 10 = highest possible level of activity regarding knee loading. The TSK uses 11 items across the domain ‘fear of movement/(re‐)injury’; it is scored using 4‐point Likert scales. Internal consistency and structural validity of the TSK were found to be sufficient [7].

RTS: A successful RTS was given when a participant achieved his/her preinjury level of sports participation as defined by the same type, frequency, intensity and quality of performance as before the injury [18]. All aspects had to be reached for the participant to be rated as ‘successfully RTS’ [18]. The (potential) RTS success was prospectively monitored, not only at predetermined time points.

### Statistical analysis

We tolerated a 5% alpha error probability for all significance testing. All statistical analyses were performed with SPSS version 28 (IBM, USA).

The sample and baseline values of the anthropometric, injury‐, sport‐ and reconstruction‐related data were described as means and standard deviations.

Potential determinates for a subsequent injury were all baseline values, traits, self‐report and objective functional outcomes as well as training and rehabilitation measures, as well as RTS success. These variables were all incorporated as (interval scaled or factorial) independent variables in the logistic mixed modelling. Three models were calculated: one for the occurrence of any subsequent total ACL re‐rupture (ipislateral or contralateral rupture), one for the occurrence of any secondary subsequent knee injury and one for any secondary subsequent problem of the lower limbs. The logistic mixed models were calculated as restricted maximum likelihood models. Models were, a priori defined, calculated with mostly fixed slopes and intercepts. Participants were modelled as a priori defined random effects. In general, a stepwise fitting procedure was employed for any of the three models. Potential contributor variables were sequentially added to the model, starting with the variable contributing the most to improving model fit. The process was continued until the inclusion of additional variables no longer resulted in a statistically significant improvement in the model fit.

As model descriptors, we calculated estimates (with their corresponding 95% CI) and odds ratios (OR) with, again, their corresponding 95% CIs. The overall classification accuracy (number of correctly predicted participants who would or would not have a subsequent injury) as well as sensitivity (number of correctly classified participants who experienced a subsequent injury) and specificity (number of correctly classified participants who did not experience a subsequent injury) were likewise calculated.

## RESULTS

### Sample description and baseline values

From the samples of the underlying studies, 259 potentially eligible persons were screened; 194 of these could be included in the present re‐analysis. Of those included, 46 dropped out due to consent withdrawal during the study conduction. For the final analysis, 148 participants were considered eligible.

Overall, eight participants suffered from a subsequent ACL injury (seven ipsilateral, one contralateral side rupture). In total, 15 participants sustained a secondary knee injury (subsequent ACL injuries: 8; meniscus tear lateral: 2; medial: 1; medial collateral ligament (MCL): 1; cartilage damage: 2; patellar ligament tear: 1), and 25 participants experienced a generalised subsequent issue of the lower limbs (subsequent knee injuries: 15; ankle distortion: 3; arthrofibrosis: 3; plica resorption: 2; cyclops: 2). The duration between the reconstruction and the secondary ACL injury ranged between 104 and 728 days (mean 503 days, standard deviation 170 days).

The anthropometric, injury‐, sport‐ and reconstruction‐related data to describe the total sample is displayed in Table 1. In addition to the description of the total sample, the data for those who experienced a subsequent ACL injury is displayed on its own.

**Table 1:** Numeric and percentage distributions of all baselines and traits.

			Total sample		Subsequent ACL injury	
Domain	Outcome (numbers)	Value/unit	Number	%	Number	%
Socio‐demographic	Sex/gender (148)	Female	63	42.6	5	62.5
		Male	83	56.1	3	37.5
		Diverse or nonbinary	2	1.4	0	0
Sport	Athletic status (140)	Nonelite	127	85.8	6	75.0
		Elite	13	8.8	2	25.0
	Tegner activity level preinjury (146)	3	13	8.8	0	0
		4	30	20.4	0	0
		5	19	13	2	25
		6	30	20.4	2	25
		7	43	29.1	2	25
		8	44	29.8	0	0
		9	51	34.5	1	12.5
		10	12	8.2	0	0
Injury	Injury mechanism (141)	Contact free	111	75.0	7	87.5
		Indirect contact	15	10.1	0	0
		Direct contact	15	10.1	1	12.5
Surgery	Graft type (tendon) (148)	Semitendinosus (+gracilis)	125	84.5	6	75.0
		Quadriceps femoris	23	15.5	2	25.0
Return to sport	Success	No	65	43.9	7	87.5
		Yes	83	56.1	1	12.5
Domain	Outcome	Unit/subscale	Mean	SD	Mean	SD
Socio‐demographic	Body mass index (148)	kg/m^2^	23.9	3.5	23.6	3.4
	Age (148)	Years	25.3	5.1	24.4	4.6
Time	Between injury and surgery (145)	Days	93	116	73	50

*Note*: The sociodemographic, sport‐, injury‐ and surgery‐specific characteristics of the study sample are shown for the total sample and subsequently separated into those who experienced a subsequent ACL injury and those who did not experience any subsequent issue.

Abbreviations: ACL, anterior cruciate ligament; SD, Standard deviation.

### Prognostic factors: Knee function as a prognostic factor

**Figure 2:**
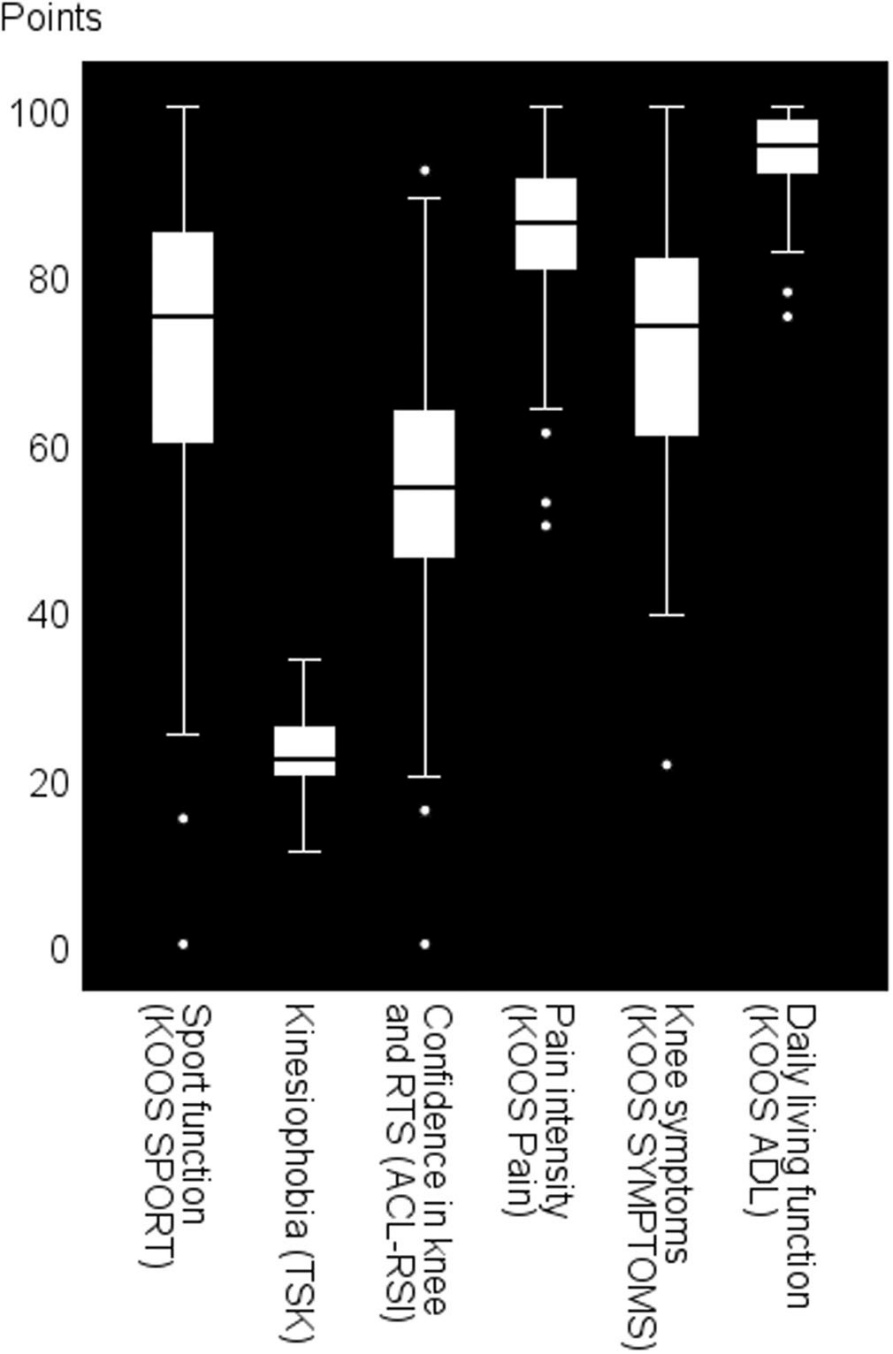
Self‐reported outcomes with a potential prognostic value for a subsequent injury. All outcome values are displayed as median, interquartile ranges (boxes), ranges (whisker bars) and outliers (dots) for the total sample of *n* = 148. ACL‐RSI, anterior cruciate ligament return to sport after injury scale name; ADL, activities of daily life; KOOS, knee injury and osteoarthritis outcome score.

All patient‐reported functional outcomes are displayed in Figure 2.

**Figure 3:**
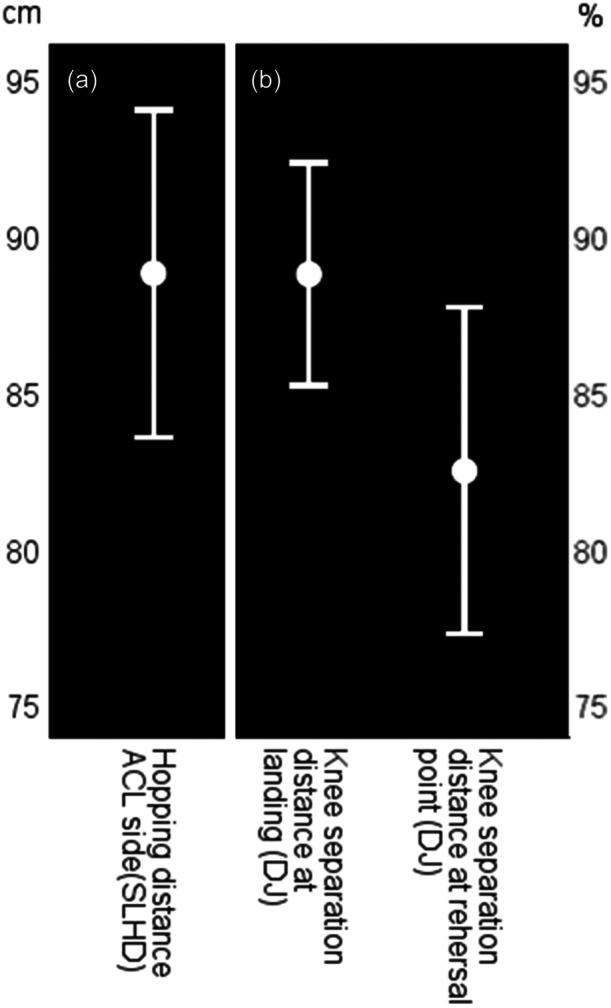
Hopping distance (left side, (a)) at the single leg hop for distance (SLHD) (anterior cruciate ligament [ACL] side only) and (b) knee separation distances at landing and at the lowest point during the drop jump (DJ) screening test. All data are displayed as means and confidence intervals for the total sample.

**Figure 4:**
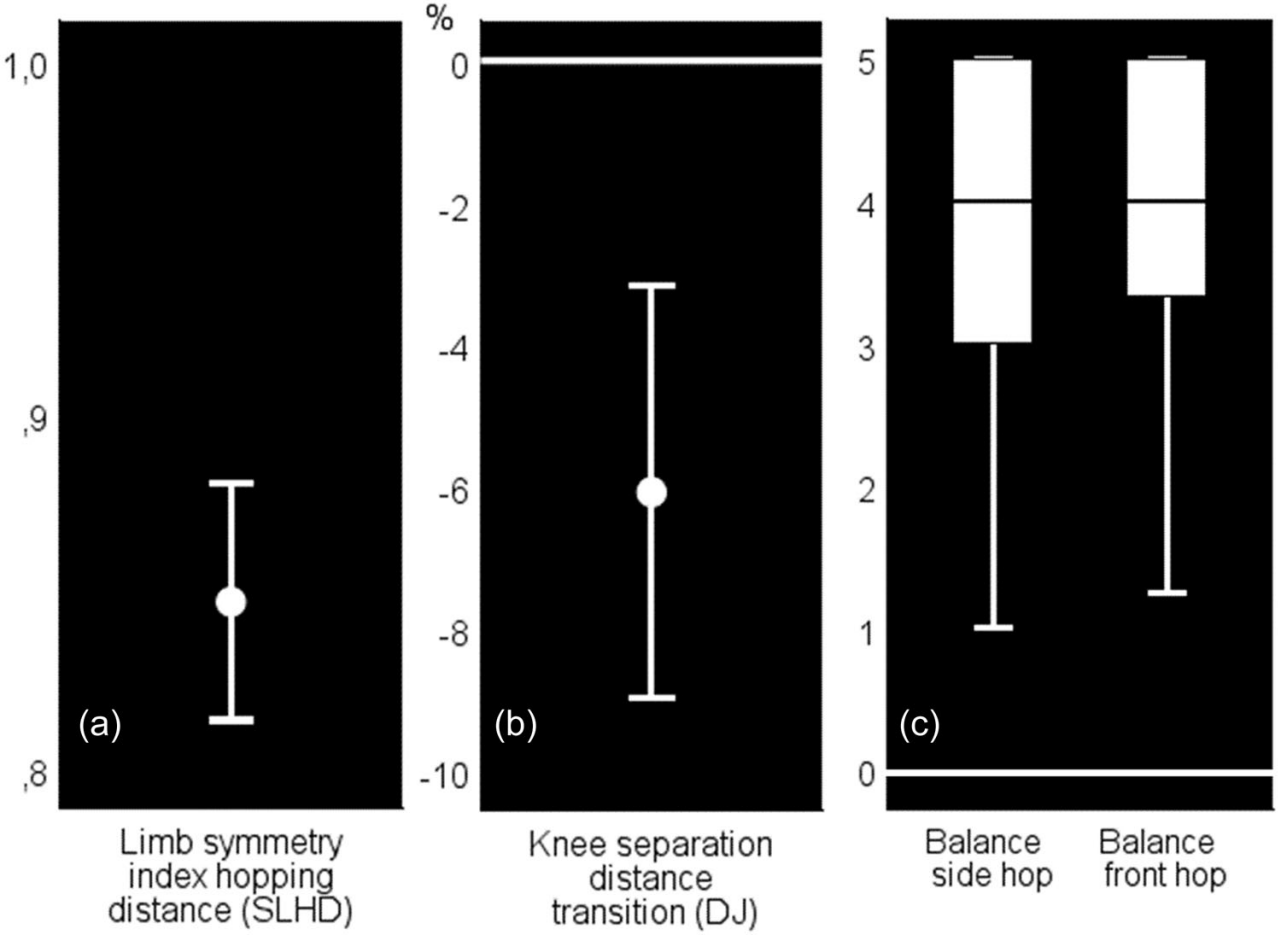
Limb symmetry index of the hopping distance at the single leg hop for distance (SLHD) (a) and transition from landing to lowest point of the knee separation distances during the drop jump (DJ) screening test (b). Data are displayed as means and confidence intervals for the total sample (c) displays the values of the balance side and front hop. Data in (c) are displayed as medians, interquartile ranges (boxes), and ranges (whisker bars).

The objectively assessed functional outcomes (hop and jump tests) assessed are displayed in Figures 3 and 4. Figure 3 displays the Single leg hop for distance for the reconstructed side as well as the knee separation distances at landing and at the lowest point during the Drop jump screening test. In Figure 4, the symmetry index of the hopping distance at the single leg hop for distance, the transition from landing to the lowest point of the knee separation distance during the drop jump screening test and the values of the Balance side and front hop are displayed.

### Determinants of a subsequent ACL injury

The final model for the predictors of a subsequent ACL injury led to a total of 98.9% of correct classifications, a sensitivity of 83.3%, and a specificity of 100% (*n* = 93 participants in the model). For any subsequent knee injury, the values were 70.0% (sensitivity) and 100% (specificity), leading to a total of 96.7% of correct classifications, whereas the probabilities to predict any follow‐up issue of the lower limbs was 66.7% and 96.2%, leading to a total of correct classifications of 90.6%.

The detailed estimates of the overall model are displayed in Table 2. The data are separated for the following dependent variables: subsequent ACL injury, any subsequent knee injury, and any subsequent issues of the lower limbs. The variables which were excluded during modelling are displayed in Supporting Information S1: Table S2.

**Table 2:** Determinates of subsequent injuries (A: subsequent ACL ruptures, B: any subsequent knee injury, C: any subsequent issue of the lower limbs) after ACL reconstruction derived by logistic mixed modelling.

	Estimate	Estimate: 95% confidence interval		Odds ratio	Odds ratio: 95% confidence interval		*p*‐Value
		Lower level	Upper level		Lower level	Upper level	
A: Subsequent ACL ruptures							
Intercept	−41.97	−85.2	1.32	0.00	0.00	3.75	0.06
Intervention group	−2.49	−6.78	1.80	0.08	0.00	6.07	0.25
Body mass index (kg × m^−1^)	0.27	−0.38	0.91	1.31	0.69	2.49	0.41
Hamstrings tendon graft	−0.74	−3.70	2.22	0.48	0.03	9.17	0.62
Quadriceps tendon graft	0.00	Reference	Reference	Reference	Reference	Reference	Reference
Knee loading level (Tegner activity scale)	3.37	0.06	6.67	29.03	1.07	791	0.05
Kinesiophobia	0.68	0.13	1.24	1.98	1.13	3.44	0.02
Knee separation distance—transition (%)	−0.23	−0.43	−0.02	0.80	0.65	0.98	0.03
Balanced side hop (points)	−2.07	−4.43	0.28	0.13	0.01	1.33	0.08
Total rehabilitation amount since reconstruction (minutes)	0.01	−0.01	0.02	1.01	0.99	1.02	0.55
RTS success	−28.3	−7324	7268	0.00	0.00	NA	0.99
Sporting function (KOOS SPORT) Cutoff fulfilled	4.80	−0.61	10.2	121.9	0.54	27,448.12	0.08
SLHD LSI cutoff success × RTS success	24.76	−7271	73,201	5.65 × 10^10^	0.00	NA	1.00
SLHD LSI cutoff success × no RTS success	4.53	−0.92	9.99	93.2	0.40	21,794	0.10
B: Any subsequent knee injury							
Intercept	1.128	−3.585	5.841	3.089	0.028	344.229	0.63
Intervention group	−0.546	−3.613	2.52	0.579	0.027	12.431	0.72
Elite athlete	−1.261	−3.822	1.3	0.283	0.022	3.669	0.33
Recreational athlete	0.00	Reference	Reference	Reference	Reference	Reference	Reference
Hamstrings tendon graft	−7.199	−13.257	−1.141	0.001	1.75 × 10^−^^10^	0.319	0.02
Quadriceps tendon graft	0.00	Reference	Reference	Reference	Reference	Reference	Reference
Total sporting activity since reconstruction (minutes)	0.005	−0.002	0.011	1.005	0.998	1.01	0.14
Total rehabilitation amount since reconstruction (minutes)	−0.01	−0.041	0.021	0.99	0.959	1.02	0.52
Kinesiophobia	0.29	0.009	0.56	1.33	1.009	1.76	0.04
Knee separation distance—transition (%)	−0.3	−0.5	−0.1	0.741	0.607	0.90	0.004
Balanced front hop (points)	−2.01	−3.37	−0.65	0.134	0.034	0.52	0.004
SLHD LSI cutoff success × RTS success	−17.9	−9555	9519	1.66E‐08	0	NA	0.997
SLHD LSI cutoff success × no RTS success	6.51	2.35	10.653	669.197	10.585	42,309	0.003
No SLHD LSI Cutoff success × RTS success	−35.5	−13,135	13,064	3.93E‐16	0	NA	0.996
No ACL RSI cutoff × RTS success	15.7	−9522	9553	6,710,738	0	NA	0.997
ACL RSI cutoff success × RTS success	−3.46	−6.579	−0.348	0.031	0.001	0.706	0.03
C: Any subsequent lower limb injury							
Intercept	−12.5	−21.2	−3.70	3.84 × 10^−^^6^	5.94 × 10^−10^	0.025	0.006
Intervention group	0.028	−2.037	2.094	1.029	0.13	8.11	0.98
Female gender	−2.46	−4.68	−0.24	0.085	0.009	0.78	0.03
Male gender	0.00	Reference	Reference	Reference	Reference	Reference	Reference
Body mass index (kg × m^−1^)	0.29	0.037	0.53	1.33	1.04	1.70	0.025
Hamstrings tendon graft	−2.41	−4.88	0.066	0.09	0.008	1.07	0.056
Quadriceps tendon graft	0.00	Reference	Reference	Reference	Reference	Reference	Reference
Knee loading level (Tegner activity scale)	0.37	−0.188	0.927	1.447	0.829	2.53	0.191
Kinesiophobia	0.124	−0.066	0.315	1.132	0.936	1.37	0.197
Balanced side hop (points)	0.262	−0.827	1.351	1.299	0.437	3.86	0.634
RTS success	−0.104	−2.435	2.228	0.902	0.088	9.28	0.93
Knee separation distance—transition (%)	−0.073	−0.146	−0.001	0.929	0.865	0.999	0.046
Sporting function (KOOS SPORT) cutoff fulfilled	−0.308	−2.018	1.401	0.735	0.133	4.059	0.721
SLHD LSI cutoff success × RTS success	−0.039	−2.272	2.194	0.962	0.103	8.969	0.972
SLHD LSI cutoff success × no RTS success	3.966	0.898	7.035	52.789	2.455	1135	0.012

*Note*: The outcome was: part A: subsequent ACL tear, part B: any subsequent knee injury and part C: any subsequent injury of the lower limbs. All variables included in the final model are displayed.

Abbreviations: ACL‐RSI, anterior cruciate ligament–return to sport after injury questionnaire; KOOS, The Knee Injury and Osteoarthritis Outcome Score; LSI, limb symmetry index; NA, not applicable; SLHD, single leg hop for distance.

The major predictors for higher likelihood of a subsequent injury were higher kinesiophobia (all models), higher knee loading levels (Tegner activity scale), lower performance levels at the Balanced hops, higher knee separation distance during the transition of landing a drop jump (subsequent ACL injury and any subsequent knee injury subsequent injury), the graft type (any subsequent knee injury only) and male gender as well as a higher BMI (any subsequent issues of the lower limbs secondary issue only).

## DISCUSSION

### Statement of principal findings and hypothesis verification

The most important findings of the present cohort study were that numerous predictors for a higher chance of a subsequent injury were revealed in. The most important outcomes with such a predictive value were: higher kinesiophobia values, higher knee loading levels, assessed by the Tegner activity scale, lower performance levels in the Balanced hops (all predictive for a subsequent ACL injury, for any subsequent knee injury and for any subsequent issue of the lower limbs), higher knee separation distance during the transitional movement of landing a drop jump (only in the models for subsequent ACL and any knee injury), the graft type (any subsequent knee injury only) and male gender as well as a higher BMI (any secondary issue only). Both our hypotheses (1) and (2) were thus verified.

### Comparison to the available evidence

We found a general classification accuracy of almost 100%, with perfect specificity and sensitivity of more than 80%. Although these values are within the same range of other test batteries that have attempted to predict a subsequent secondary injury of the ACL, our values are slightly better. For example, the total classification accuracy was 90.9%, sensitivity was 75% and specificity was 93.4% in a comparable study [1]. Numerous factors we have identified correspond well with factors which are believed to increase the risk of graft failure and/or a re‐rupture after an ACL reconstruction described in this and in a few earlier studies: return to a high activity level/sport [33], general or early RTS [1, 26], hamstring tendon (HT) autografts (vs. bone–patellar tendon–bone [BPTB] autografts) [33], the confidence to RTS [1], gender/sex [4, 33] and lower BMI [1, 33]. The ORs for BMI (although not significant in our model to predict a second ACL injury) are within a comparable margin, an OR of slightly >1.0 is specified in the literature [1, 33], the same is true for returning to a higher level of sporting activity (OR of 2.03 [33] to OR of 3.26 [4]). Gender was, in our prediction models, only relevant to predict any secondary issue of the lower limbs but not in the ACL or knee models. This is also in agreement with other findings. In some studies, no impact of sex was observed for total second ACL injury risk or contralateral ACL injury risk [25], while other found that females have a lower risk for a subsequent ipsilateral and a higher risk for a subsequent contralateral secondary ACL injury compared with males [4, 33].

In contrast, a few potential predictors which had no impact on our cohort are described in the corresponding literature receiving reconstructive surgery within 1 month [16] or within 3 months post injury [4], for example. In our study, the time between injury and surgery did not contribute to any secondary injury risk. Further factors where this applies are concomitant remaining MCL instabilities [33] and age (younger age might contribute both to a general [16] and contralateral secondary ACL injury risk [4]). Again, these findings could not be reproduced in the present study. Rehabilitation‐associated measures are another issue to discuss. Starting rehabilitative measures earlier (i.e., within 3 days postsurgery) is reported to lead to a reduction of the re‐injury risk [16], while a decreased number of rehabilitation sessions increased re‐injury risk [16]. In our study, the amount of rehabilitation was (in all models) a relevant contributor which increased the model quality but lacked a significant contribution. In general, the total amount of rehabilitative measures may reduce the risk for a subsequent injury after an index ACL reconstruction, whereas the total amount of sports activity may have increased it. The latter may be a result of the associated increased exposure time. This would correspond to the specified increased risk in association with higher sport levels in both our models and in the literature.

We omitted to assess a few potential predictors during the study conduction. Family history of an ACL injury, higher posterolateral tibial slope, preoperative high‐grade anterior knee laxity, higher baseline Marx activity level and smaller graft diameter might also contribute to an increased risk of a subsequent ACL injury [4, 33]. Further potentially relevant functional contributors are, for example, hamstring strength side symmetry and the hamstrings/quadriceps strength ratio [1]. In our trial, we have not assessed muscle strength. This must be considered as a limitation. However, numerous other functional outcomes were assessed in the present study, of which the Balanced hops function and the knee separation distance during the transition of landing a Drop jump predicted future secondary ACL and any knee subsequent injuries. This corresponds well to the findings of a current systematic review, which found ‘a combination of isokinetic quadriceps strength at different velocities and a number of hop tests, plus self‐reported outcomes as predictive with various effect sizes’ [2]. The self‐reported outcomes mentioned in the review fit well with our findings. Beyond that, we found a major contribution of kinesiophobia (all models) and, to a lesser extent, also of psychological confidence to RTS (ACL‐RSI values) and self‐reported sporting function (KOOS Sport) to the risk of re‐injury. Less kinesiophobia, more confidence and better self‐reported functional values were associated with a decrease in injury risk.

With a subsequent re‐injury rate of 5%, our sample ranks at the bottom of comparable samples. A total secondary ACL injury risk of 15%–22% [31] is usually specified [24], with equal contribution of ispi‐ and contralateral secondary injuries [24]. All studies which analysed comparative studies used to discuss our predictors show higher re‐injury rates than our sample as well [1, 13, 16, 33]. Reasons for these comparably lower values may be related to the preselection of certified knee surgeons and the guided and progressive rehabilitation and RTS protocols adopted in the present sample (although no relevant differences occurred between those who received a tailored re‐injury‐preventive intervention and those who did not) [20, 22]. In contrast, the lower numbers might also result from the large extent nonelite level participants (lower exposure time) and the lack of inclusion of younger age groups (below 18 years of age).

### Practical relevance and future studies

Many of the predictors of a subsequent injury after an index ACL rupture and reconstruction are modifiable. Kinesiophobia, higher knee loading levels and the level of sporting activity, lower performance levels at qualitative hops, higher knee separation distance during the transition of landing a drop jump, and lastly, BMI, can all be modified. These contributing factors (and a few more relevant outcomes such as tibial slope and quadriceps strength) could be assessed to create an individual secondary injury risk‐profile after an ACL reconstruction. As numerous variables with a predictive value for a subsequent injury after index ACL tears are modifiable, their modification might decrease secondary injury risk. Many of the variables, particularly self‐reported and objectively assessed function, are influenceable by adequate training, which could be applied as re‐injury preventive rehabilitation. For example, functional capacities are trainable adopting late‐stage neuromuscular performance enhancing intervention in both hamstrings‐ and quadriceps‐grafted individuals [20, 22].

### Limitations

Our study is not free of limitations. The data were collected and pooled from an interventional and from a cohort trial. Still, a considerably small sample size resulted; we have further not performed an a priori sample size estimation. RTS success was recorded on a self‐report basis. This was made due to organisational issues such as different types and levels of sport resulting in a disproportionate effort to assess the success objectively. Certain outcomes, such as the intensity and quality of performance would have been more valid if an objective rating could have been used.

As half of the participants whose knees were reconstructed with a hamstring graft (as a part of the intervention trial) and all of the participants whose knees were reconstructed with a quadriceps graft (as a part of the matching cohort) performed a specific (and not the Usual care) re‐injury preventive program, which may have led to certain interactions among the independent variables. Beyond the re‐injury preventive program, we only know the superficial training specifics and loads, but not the quality of the rehabilitation measures, such as the coaching strategies. These approaches may differ between the participants. Furthermore, our sample showed only a few secondary injuries and we able to follow‐up only 55% of the original cohort. Both limits the power of the analyses.

## CONCLUSION

The most important predictors of a subsequent injury after an index ACL rupture and reconstruction were higher kinesiophobia, higher knee loading levels, higher levels of sporting activity, lower performance levels at qualitative hops, higher knee separation distance during the landing a drop jump, being male and having a higher BMI. These predictors, together with other important outcomes such as tibial slope and quadriceps strength, could be assessed to create an individual risk profile after an ACL reconstruction. The modification of some or all of these variables might decrease the secondary injury risk after the index ACL rupture. Many of the variables, in particular self‐reported and objectively assessed function, are influenceable by adequate training methods. Consequently, future work is requested to investigate whether the modification of modifiable predictors of a second ACL injury indeed lowers re‐injury risk.

## AUTHOR CONTRIBUTIONS

All authors contributed to the manuscript and released the final version. For details, please refer to the submission system.

## CONFLICT OF INTEREST STATEMENT

The authors declare no conflicts of interest.

## ETHICS STATEMENT

Ethics Committee of the Hessen Regional Medical Council (reference approval no. FF 104/2017). Date of the final approval of the study protocol was June 27, 2018. Patients were involved in the development of the intervention programme but not in the design or conduct of the study, nor in the outcome selection. Formal written informed consent was obtained from all participants prior to inclusion by means of a structured informed consent process. All participants signed written and gave informed oral consent and further provided consent to their data being re‐analysed in secondary analyses prior to enrolment.

## Supporting information

STROBE‐checklist‐v4.

Supplement PReP8.

## Data Availability

The full dataset will be made available in a public repository when all project‐wide studies and planned re‐analyses are published. Until then, data are available upon request.
