# Supplementary material for: Jump performance and hop function, kinesiophobia and return to sports are important prognostic factors for a subsequent injury after an anterior cruciate ligament reconstruction: A 2‐year follow‐up cohort study
Source: Knee Surg Sports Traumatol Arthrosc. 2025 Oct 27;34(1):338–50. doi: 10.1002/ksa.70104 (PMC12747599; doi:10.1002/ksa.70104)
Supplement: Supplementary file 2 — Supplement PReP8. [file KSA-34-338-s001.docx]

Supplemental files for

Jump and hop performance, kinesiophobia, knee confidence, and return to sports are important prognostic factors for a subsequent injury after an anterior cruciate ligament reconstruction: A 2-year follow-up cohort study

Supplemental Table 1: Training regimen ^1,2^. The programme (Stop-X, in German) can be found online (https://www.stop-x.de/ - permanent link status as of 12 April, 2024).

| Wound healing phase or time post-surgery | Therapy goal | Usual care | | Stop-X |
| --- | --- | --- | --- | --- |
| *Inflammation phase: initial* | *Reduction of the inflammation* | *Brace to restrict range of motion, restriction 0-0-90°*  *20 kg partial knee load* | | |
|  |  | *Manual therapeutic: Patella mobilisation, passive knee joint closed kinematic chain mobilisation, hip and ankle integrated* | | |
|  |  | *Measures against post-surgery swelling and temperature increase home-based measures for auto-mobilisation (under compression)* | | |
|  |  | *Trunk and scapula control* *non-affected side static motor control, i.e. single leg stance* | | |
| *Proliferation phase:*  ~*week 3 -12 post-reconstruction* | *Pain reduction and mobilisation* | *Manual therapeutic: Knee joint mobilisation in restricted RoM (Ext, Flex) Patella mobilisation* | | |
|  |  | *Stretching: M. ischiocruales, M. gastrocnemius, M. iliopsoas, M. tractus iliotibialis* | | |
|  |  | *Motor control exercises for trunk and scapula function non-affected side static motor control, i.e. single leg stance* | | |
| ~*after week 7 post-reconstruction* |  | *Onset motor control training* | | |
|  |  | *Open kinetic (dorsal only) chain* | | |
| ~*after week 10 post-reconstruction* |  | *Corrective exercise with the aim of preparing for RTS level I* | | |
|  |  | *Single-leg dynamic stabilising/motor control exercises* | | |
|  |  | *Eccentric knee mobilisation exercises open frontal kinetic chain* | | |
| Remodelling phase  Level I function: ~after week 13 post-reconstruction | Functional enhancement and recurrence prevention  Dynamic balance  Closed chain strength  Open chain strength  Sagittal plane jumps  Other planes jump  Change of direction and cutting manoeuvre | Closed and open kinetic chain (ventral and dorsal) | Information, risk assessment, correction of risky movement characteristics  teaching of basic preventive strategies  total programme at/after RTS clearance  Running exercises/agility exercises  Straight-on running, hip external rotation, change of direction runs  Running with sagittal plane distance and vertical jump components.  Self-perturbed balance exercises  Single leg stance with (individually and combined) ball bouncing/unstable surface/passing a ball  Jumping exercises/plyometrics, vertical and distance  Strengthening exercises (own body weight and home-available loads such as bottles)  Russian Hamstrings  Dynamic and side planks with/without heel raising  Hip abduction  Onelegged squats | |
|  |  | Strength endurance training for (in particular) the ischio-crural muscles |  |  |
|  |  | Dynamic stabilising motor control exercises, including Jump landings on unstable surfaces |  |  |
| Level II function: ~months 4-6 post-reconstruction |  | Dynamic stabilising motor control exercises, including single (affected) leg jump landings on unstable surfaces |  |  |
|  |  | Onset impact exercises |  |  |
| Level III function: ~after month 7 post-reconstruction |  | Dynamic exercises (frontal plane) |  |  |
|  |  | Side cutting manoeuvres |  |  |
| Level IV function: not before 10^th^ month post-reconstruction |  | Dynamic multi-directional stabilisation |  |  |

1. Supplemental Table 2: Excluded variables during the subsequent injury modelling

| A: subsequent ACL ruptures | Estimate | Estimate: 95% confidence interval | | Odds ratio | Odds ratio: 95% confidence interval | | p-value |
| --- | --- | --- | --- | --- | --- | --- | --- |
|  |  | Lower level | Upper level |  | Lower level | Upper level |  |
| Intercept | 4.01 | -6.537 | 14.556 | 55.132 | 0.001 | 2097243.256 | 0.452 |
| Confidence in RTS (ACL-RSI) | 0.004 | -0.053 | 0.061 | 1.004 | 0.948 | 1.063 | 0.888 |
| Pain intensity (KOOS) | 0.037 | -0.098 | 0.173 | 1.038 | 0.906 | 1.189 | 0.585 |
| Symptoms (KOOS) | -0.01 | -0.084 | 0.064 | 0.99 | 0.919 | 1.066 | 0.791 |
| ADL function (KOOS) | -0.081 | -0.267 | 0.104 | 0.922 | 0.766 | 1.11 | 0.387 |
| Balance front hop [points] | -0.669 | -1.533 | 0.195 | 0.512 | 0.216 | 1.215 | 0.127 |
| Total sporting activity since reconstruction [minutes] | 0.006 | 0 | 0.012 | 1.006 | 1 | 1.012 | 0.036 |
| Total of rehabilitation since reconstruction [minutes] | -0.004 | -0.025 | 0.018 | 0.996 | 0.975 | 1.018 | 0.731 |
| Intercept | -1.668 | -5.477 | 2.142 | 0.189 | 0.004 | 8.515 | 0.388 |
| Male gender | -0.681 | -2.205 | 0.844 | 0.506 | 0.11 | 2.325 | 0.378 |
| Female gender | reference | reference | reference | reference | reference | reference | reference |
| Age [years] | -0.029 | -0.176 | 0.118 | 0.972 | 0.839 | 1.125 | 0.698 |
| Elite athlete | 1.338 | -0.628 | 3.305 | 3.812 | 0.533 | 27.245 | 0.18 |
| Recreational athlete | reference | reference | reference | reference | reference | reference | reference |
| Contact injury mechanism | -0.345 | -2.795 | 2.106 | 0.708 | 0.061 | 8.214 | 0.781 |
| Indirect contact injury mechanism | -16.016 | -5757.672 | 5725.641 | 1.11E-07 | 0 | NA | 0.996 |
| No contact injury mechanism | reference | reference | reference | reference | reference | reference | reference |
| Time between injury and reconstruction [days] | -0.001 | -0.009 | 0.007 | 0.999 | 0.991 | 1.007 | 0.84 |
| B: Any subsequent knee injury | | | | | | | |
| Intercept | -3.583 | -8.135 | 0.969 | 0.028 | 0 | 2.636 | 0.122 |
| Male gender | -0.313 | -1.507 | 0.88 | 0.731 | 0.221 | 2.412 | 0.604 |
| Female gender | 0 | . | . | . | . | . | . |
| Body mass index [kg*m-1] | 0.011 | -0.152 | 0.174 | 1.011 | 0.859 | 1.19 | 0.892 |
| Age | 0.068 | -0.037 | 0.173 | 1.071 | 0.964 | 1.189 | 0.2 |
| Contact injury mechanism | -0.699 | -2.826 | 1.429 | 0.497 | 0.059 | 4.174 | 0.517 |
| Indirect contact injury mechanism | -15.423 | -2982.359 | 2951.514 | 2.01E-07 | 0 | . | 0.992 |
| No contact injury mechanism | reference | reference | reference | reference | reference | reference | reference |
| Time between injury and reconstruction [days] | -0.002 | -0.008 | 0.005 | 0.998 | 0.992 | 1.005 | 0.637 |
| Intercept | -3.897 | -14.065 | 6.27 | 0.02 | 7.79E-07 | 528.543 | 0.448 |
| Knee loading level (Tegner activity scale) | 0.19 | -0.221 | 0.601 | 1.209 | 0.802 | 1.825 | 0.36 |
| Knee function during sport (KOOS) | -0.015 | -0.067 | 0.037 | 0.985 | 0.935 | 1.037 | 0.562 |
| Pain intensity (KOOS) | -0.011 | -0.128 | 0.106 | 0.989 | 0.88 | 1.112 | 0.853 |
| Symptoms (KOOS) | -0.012 | -0.074 | 0.051 | 0.988 | 0.928 | 1.052 | 0.707 |
| ADL function (KOOS) | 0.036 | -0.125 | 0.196 | 1.036 | 0.883 | 1.216 | 0.66 |
| Balance side hop [points] | -0.03 | -0.774 | 0.714 | 0.971 | 0.461 | 2.042 | 0.937 |
| Total intervention activity since reconstruction [minutes] | 0.004 | -0.001 | 0.009 | 1.004 | 0.999 | 1.009 | 0.146 |
| C: Any subsequent lower limb injury | | | | | | | |
| Intercept | -5.397 | -9.387 | -1.408 | 0.005 | 8.38E-05 | 0.245 | 0.008 |
| Body mass index [kg*m-1] | 0.098 | -0.028 | 0.223 | 1.103 | 0.973 | 1.25 | 0.125 |
| Age | 0.069 | -0.024 | 0.161 | 1.071 | 0.977 | 1.175 | 0.143 |
| Elite athlete | 1.354 | -0.161 | 2.869 | 3.873 | 0.852 | 17.611 | 0.079 |
| Recreational athlete | reference | reference | reference | reference | reference | reference | reference |
| Contact injury mechanism | -0.923 | -2.681 | 0.835 | 0.397 | 0.068 | 2.305 | 0.301 |
| Indirect contact injury mechanism | -0.27 | -1.973 | 1.434 | 0.763 | 0.139 | 4.194 | 0.754 |
| No contact injury mechanism | reference | reference | reference | reference | reference | reference | reference |
| ZeitVerlOP | -0.002 | -0.008 | 0.004 | 0.998 | 0.992 | 1.004 | 0.471 |
| Intercept | -0.213 | -8.235 | 7.81 | 0.808 | 0 | 2464.493 | 0.958 |
| Confidence in RTS (ACL-RSI) | -0.013 | -0.055 | 0.029 | 0.987 | 0.946 | 1.029 | 0.528 |
| Pain intensity (KOOS) | -0.04 | -0.14 | 0.059 | 0.96 | 0.869 | 1.061 | 0.421 |
| Symptoms (KOOS) | 0.03 | -0.026 | 0.085 | 1.03 | 0.975 | 1.088 | 0.289 |
| ADL function (KOOS) | 0.013 | -0.121 | 0.146 | 1.013 | 0.886 | 1.157 | 0.851 |
| Balance front hop [points] | -0.359 | -0.98 | 0.262 | 0.698 | 0.375 | 1.299 | 0.253 |
| Total intervention activity since reconstruction [minutes] | 0.011 | -0.002 | 0.024 | 1.011 | 0.998 | 1.024 | 0.085 |
| Total sporting activity since reconstruction [minutes] | 0.002 | -0.004 | 0.009 | 1.002 | 0.996 | 1.009 | 0.478 |
| Total of rehabilitation since reconstruction [minutes] | 0.003 | -0.009 | 0.016 | 1.003 | 0.991 | 1.016 | 0.618 |

KOOS, The Knee Injury and Osteoarthritis Outcome Score; ACL-RSI, Anterior cruciate ligament – return to sport after injury questionnaire; SLHD, single leg hop for distance; LSI, limb symmetry index; NA, not applicable

References

1. Niederer D, Keller M, Schuttler KF, et al. Late-stage rehabilitation after anterior cruciate ligament reconstruction: A multicentre randomised controlled trial (PReP). *Annals of Physical and Rehabilitation Medicine*. 2023;67(4):101827.

2. Niederer D, Keller M, Achtnich A, et al. Effectiveness of a home-based re-injury prevention program on motor control, return to sport and recurrence rates after anterior cruciate ligament reconstruction: study protocol for a multicenter, single-blind, randomized controlled trial (PReP). *Trials*. 2019;20(1):495. doi:10.1186/s13063-019-3610-2
